# Supplementary material for: General practitioners’ perspectives on statutory skin cancer screening–A questionnaire-based cross-sectional survey in Germany
Source: PLoS One. 2024 Aug 8;19(8):e0308508. doi: 10.1371/journal.pone.0308508 (PMC11309404; doi:10.1371/journal.pone.0308508)
Supplement: S1 Appendix — Report according to the requirements of the STROBE guidelines (https://www.strobe-statement.org/). (DOCX) [file pone.0308508.s001.docx]

STROBE Statement—checklist of items that should be included in reports of observational studies

|  | Item No. | Recommendation | Page  No. | | Relevant text from manuscript |
| --- | --- | --- | --- | --- | --- |
| **Title and abstract** | 1 | (*a*) Indicate the study’s design with a commonly used term in the title or the abstract | p. 1 | a questionnaire-based cross-sectional survey in Germany | |
|  |  | (*b*) Provide in the abstract an informative and balanced summary of what was done and what was found | p. 2 |  | |
| Introduction | | | |  | |
| Background/rationale | 2 | Explain the scientific background and rationale for the investigation being reported | p. 3f. |  | |
| Objectives | 3 | State specific objectives, including any prespecified hypotheses | p. 3f. | The aim of this survey was to assess how GPs conduct the screening and provide patient education in this context. We investigated whether there were differences between regions (federal state, location of physician´s office), professional experience (professional experience in years, number of monthly screenings, age) and gender. In addition, we explored the personal opinion of GPs about the SCS programme in general, as well as uncertainties and barriers to the implementation. | |
| Methods | | | |  | |
| Study design | 4 | Present key elements of study design early in the paper | p. 4f. | section “Study design” & “Survey” | |
| Setting | 5 | Describe the setting, locations, and relevant dates, including periods of recruitment, exposure, follow-up, and data collection | p. 4f. | sections “Study design”, “Participants”, “Survey” | |
| Participants | 6 | (*a*) *Cohort study*—Give the eligibility criteria, and the sources and methods of selection of participants. Describe methods of follow-up  *Case-control study*—Give the eligibility criteria, and the sources and methods of case ascertainment and control selection. Give the rationale for the choice of cases and controls  *Cross-sectional study*—Give the eligibility criteria, and the sources and methods of selection of participants | p. 4 | Contact details of GPs with permission to perform SCS were obtained from the Associations of Statutory Health Insurance Physicians in Saxony and Bavaria. All physicians practicing general medicine at that time who were authorized to perform SCS and were active in the study region were included. GPs provided written informed consent to participate. A predefined ID was used to ensure that each GP contacted could only participate in the survey once. | |
|  |  | (*b*) *Cohort study*—For matched studies, give matching criteria and number of exposed and unexposed  *Case-control study*—For matched studies, give matching criteria and the number of controls per case | na |  | |
| Variables | 7 | Clearly define all outcomes, exposures, predictors, potential confounders, and effect modifiers. Give diagnostic criteria, if applicable 🡪 na | p. 5f. | sections “survey”, “quantitative analysis”, “qualitative analysis” (the outcomes are described; see also subgroup analysis) | |
| Data sources/ measurement | 8* | For each variable of interest, give sources of data and details of methods of assessment (measurement). Describe comparability of assessment methods if there is more than one group | p. 5 | section “survey” (source of data = survey, measurement = 5-point Likert Scale) | |
| Bias | 9 | Describe any efforts to address potential sources of bias | p. 14 | Potential bias is described in section “Strength and Limitations” | |
| Study size | 10 | Explain how the study size was arrived at | p. 4 | Contact details of GPs with permission to perform SCS were obtained from the Associations of Statutory Health Insurance Physicians in Saxony and Bavaria. All physicians practicing general medicine at that time who were authorized to perform SCS and were active in the study region were included. | |

Continued on next page

| Quantitative variables | 11 | Explain how quantitative variables were handled in the analyses. If applicable, describe which groupings were chosen and why | p. 5f., S2 Appendix | section “qualitative analysis”, categories in S2 Appendix |
| --- | --- | --- | --- | --- |
| Statistical methods | 12 | (*a*) Describe all statistical methods, including those used to control for confounding | p. 5 | section “Quantitative analysis” |
|  |  | (*b*) Describe any methods used to examine subgroups and interactions | p. 5 | section “Quantitative analysis” |
|  |  | (*c*) Explain how missing data were addressed | p. 5 | section “Quantitative analysis” (Missing data were explicitly stated for each result) |
|  |  | (*d*) *Cohort study*—If applicable, explain how loss to follow-up was addressed  *Case-control study*—If applicable, explain how matching of cases and controls was addressed  *Cross-sectional study*—If applicable, describe analytical methods taking account of sampling strategy | p. 4 | Section “Participants” (all GPs with permission to perform SCS who were listed in the Associations of Statutory Health Insurance Physicians in Saxony and Bavaria) |
|  |  | (*e*) Describe any sensitivity analyses | na |  |
| Results | | | | |
| Participants | 13* | (a) Report numbers of individuals at each stage of study—eg numbers potentially eligible, examined for eligibility, confirmed eligible, included in the study, completing follow-up, and analysed |  |  |
|  |  | (b) Give reasons for non-participation at each stage | p. 14 | section “strength and limitations” |
|  |  | (c) Consider use of a flow diagram | na |  |
| Descriptive data | 14* | (a) Give characteristics of study participants (eg demographic, clinical, social) and information on exposures and potential confounders | p. 6 | section “study population” & Table 1 |
|  |  | (b) Indicate number of participants with missing data for each variable of interest | p. 6-8 |  |
|  |  | (c) *Cohort study*—Summarise follow-up time (eg, average and total amount) | na |  |
| Outcome data | 15* | *Cohort study*—Report numbers of outcome events or summary measures over time | na |  |
|  |  | *Case-control study—*Report numbers in each exposure category, or summary measures of exposure | na |  |
|  |  | *Cross-sectional study—*Report numbers of outcome events or summary measures | p. 6ff | section “Results” |
| Main results | 16 | (*a*) Give unadjusted estimates and, if applicable, confounder-adjusted estimates and their precision (eg, 95% confidence interval). Make clear which confounders were adjusted for and why they were included | p. 6ff | section “Results” (unadjusted estimates) |
|  |  | (*b*) Report category boundaries when continuous variables were categorized | na |  |
|  |  | (*c*) If relevant, consider translating estimates of relative risk into absolute risk for a meaningful time period | na |  |

Continued on next page

| Other analyses | 17 | Report other analyses done—eg analyses of subgroups and interactions, and sensitivity analyses | p. 8f. | section “subgroup analysis” |
| --- | --- | --- | --- | --- |
| Discussion | | | | |
| Key results | 18 | Summarise key results with reference to study objectives | p. 11-14 | section “Discussion” + “conclusion” |
| Limitations | 19 | Discuss limitations of the study, taking into account sources of potential bias or imprecision. Discuss both direction and magnitude of any potential bias | p. 14 | section “strength and limitations” |
| Interpretation | 20 | Give a cautious overall interpretation of results considering objectives, limitations, multiplicity of analyses, results from similar studies, and other relevant evidence | p. 11-14 | section “Discussion” + “strength & limitations” |
| Generalisability | 21 | Discuss the generalisability (external validity) of the study results | p. 11-14 | Section “Discussion”, especially comparison with previous studies on the implementation of skin cancer screening (p. 12) and potential bias (p. 14) |
| Other information | |  | | |
| Funding | 22 | Give the source of funding and the role of the funders for the present study and, if applicable, for the original study on which the present article is based | p. 4 & information on Financial Disclosure | section “study design” |

*Give information separately for cases and controls in case-control studies and, if applicable, for exposed and unexposed groups in cohort and cross-sectional studies.

**Note:** An Explanation and Elaboration article discusses each checklist item and gives methodological background and published examples of transparent reporting. The STROBE checklist is best used in conjunction with this article (freely available on the Web sites of PLoS Medicine at http://www.plosmedicine.org/, Annals of Internal Medicine at http://www.annals.org/, and Epidemiology at http://www.epidem.com/). Information on the STROBE Initiative is available at www.strobe-statement.org.
