# Supplementary material for: General practitioners’ perspectives on statutory skin cancer screening–A questionnaire-based cross-sectional survey in Germany
Source: PLoS One. 2024 Aug 8;19(8):e0308508. doi: 10.1371/journal.pone.0308508 (PMC11309404; doi:10.1371/journal.pone.0308508)
Supplement: S2 Appendix — Survey on skin cancer screening from the perspective of general practitioners. (DOCX) [file pone.0308508.s002.docx]

**S2 Appendix. Questionnaire.** Survey on skin cancer screening from the perspective of general practitioners.

| 1. **Questions about implementation of skin cancer screening and physician-patient-communication** | **Always** | **Often** | **Occasioanlly** | **Rarely** | **Never** |
| --- | --- | --- | --- | --- | --- |
|  |  |  |  |  |  |
| I **inform my patients** as part of skin cancer screening… | | | | | |
| ...about **causes** of skin cancer | □ | □ | □ | □ | □ |
| …about **prevention** of skin cancer | □ | □ | □ | □ | □ |
| …about the possible **risk of excision** of harmless skin lesions (=**false-positive** results) | □ | □ | □ | □ | □ |
| …about the risk of skin lesions being diagnosed as harmless when they are not (=**false-negative** results) | □ | □ | □ | □ | □ |
| …about the possible discovery and treatment of a skin cancer that might not have affected them later in life (=**potential overdiagnosis**) | □ | □ | □ | □ | □ |
| …about the **benefit** of skin cancer screening | □ | □ | □ | □ | □ |
| I take a **detailed medical history** (e.g. as a medical history questionnaire in the waiting room) on skin cancer risk and ask about... | | | | | |
| …personal medical history | □ | □ | □ | □ | □ |
| …family medical history | □ | □ | □ | □ | □ |
| I **examine** the following **body parts** as part of skin cancer screening.... | | | | | |
| …head/scalp | □ | □ | □ | □ | □ |
| …ears | □ | □ | □ | □ | □ |
| …mouth/oral mucosa | □ | □ | □ | □ | □ |
| …upper body | □ | □ | □ | □ | □ |
| …axillary and inguinal area | □ | □ | □ | □ | □ |
| …arms | □ | □ | □ | □ | □ |
| …hands | □ | □ | □ | □ | □ |
| …between the fingers | □ | □ | □ | □ | □ |
| …genitalia | □ | □ | □ | □ | □ |
| …buttocks | □ | □ | □ | □ | □ |
| …intergluteal fold | □ | □ | □ | □ | □ |
| …legs | □ | □ | □ | □ | □ |
| …feet | □ | □ | □ | □ | □ |
| …between the toes | □ | □ | □ | □ | □ |

| 1. **Questions about the skin cancer training programme**   In order to be able to offer skin cancer screening in your office, you have participated in an eight-hour continuing training programme accredited by the Association of Statutory Health Insurance Physicians. Please rate the following statements about the training programme! | **Strongly agree** | **Agree** | **Neutral** | **Disagree** | **Strongly disagree** |
| --- | --- | --- | --- | --- | --- |
| The training content is **relevant for** my **daily practice**. | □ | □ | □ | □ | □ |
| I see a need for regular **refresher courses**. | □ | □ | □ | □ | □ |
| If you see a need, at what intervals should refresher courses be offered? | | | | | |
| I feel **adequately trained** to perform skin cancer training. | □ | □ | □ | □ | □ |
| I feel **confident** in performing skin cancer screening. | □ | □ | □ | □ | □ |
| If you feel unconfident, please explain: | | | | | |
| I would need more knowledge in my daily practice for ... | | | | | |

1. **Questions about your personal attitude towards skin cancer screening**

Since 2008, the standardized screening examination for skin cancer has been covered by all statutory health insurers for insured persons aged 35 and older at two-year intervals. Please rate the following statement:

**I think skin cancer screening should be a health insurance benefit at any age.**

| 🞏 Strongly agree | 🞏 Agree | 🞏 Neutral | 🞏 Disagree | 🞏 Strongly disagree |
| --- | --- | --- | --- | --- |

"An algorithm assesses skin tumors more accurately than dermatologists in a study," the National Center for Tumor Diseases in Heidelberg recently reported. Please rate the following statement:

**I am positive about the use of artificial intelligence (AI) in skin cancer screening.**

| 🞏 Strongly agree | 🞏 Agree | 🞏 Neutral | 🞏 Disagree | 🞏 Strongly disagree |
| --- | --- | --- | --- | --- |

1. **Personal Data**

| **Please state your gender:** | 🞏 Male 🞏 Female 🞏 Divers |
| --- | --- |

| **Please state your age:** | years |
| --- | --- |

**Where is your office located?**

| 🞏 | In a big city | 🞏 | In a city | 🞏 | In a rural area |
| --- | --- | --- | --- | --- | --- |
|  | (≥ 100.000 inhabitants) |  | (≥ 5.000 inhabitants) |  | (< 5.000 inhabitants) |

**When did you attend the skin cancer screening training?**

**Please answer as accurately as possible.**

Please provide the dates in month/year format (e.g. 02/2018). __ / ____

**How many skin cancer screenings do you personally perform per month?**

🞏 less than 5 🞏 between 5 and 10 🞏 more than 10

**How long have you been working as general practitioner?**

🞏 less than 5 years 🞏 5 to 15 years 🞏 more than 15 years

**Finally, is there anything you would like to add?**

We would like to give you the opportunity to address topics that are important to you in connection with skin cancer screening (e.g. **payment**, **training programme**...).
