# Supplementary material for: General practitioners’ perspectives on statutory skin cancer screening–A questionnaire-based cross-sectional survey in Germany
Source: PLoS One. 2024 Aug 8;19(8):e0308508. doi: 10.1371/journal.pone.0308508 (PMC11309404; doi:10.1371/journal.pone.0308508)
Supplement: S4 Appendix — Categories of qualitative analysis generated from the responses of general practitioners. (DOCX) [file pone.0308508.s004.docx]

**S3 Appendix. Categories.** Categories of qualitative analysis generated from the responses of General Practitioners.

| **Category 1: Uncertainty when performing SCS** | | |
| --- | --- | --- |
| **Code** | **Subcategory** | **Frequency** |
| C1.1 | Reasons for uncertainties | 34 |
| C.1.1.1 | Differential diagnosis | 8 |
| C.1.1.2 | Lack of practical experience/routine | 8 |
| C.1.1.3 | Use of dermatoscope unclear | 7 |
| C.1.1.4 | Lack of feedback from dermatologists | 2 |
| C.1.1.5 | Missing second opinion | 2 |
| C.1.1.6 | Training content not sufficiently relevant to practice | 2 |
| C.1.1.7 | Melanoma diagnostics | 1 |
| C.1.1.8 | Localization in the genital area | 1 |
| C.1.1.9 | General diagnosis | 1 |
| C.1.1.10 | False negative findings | 1 |
| C.1.1.11 | No participation in refresher course due to overload | 1 |
| C.1.2 | Behavior in case of uncertainty | 12 |
| C.1.2.1 | Referral to specialist in case of unclear findings | 10 |
| C.1.2.2 | Use of dermatoscope in case of uncertainty | 2 |
| **Category 2: Knowledge required for SCS** | | |
| **Code** | **Category** | **Frequency** |
| C.2.1 | Dermatoscopy | 15 |
| C.2.2 | Differential diagnosis | 13 |
| C.2.2.1 | Differential diagnosis, not specified | 2 |
| C.2.2.2 | Malignancy criteria | 3 |
| C.2.2.3 | Melanocytic nevi | 2 |
| C.2.2.4 | Non-melanoma skin cancer | 2 |
| C.2.2.5 | Benign skin lesions | 1 |
| C.2.2.6 | Differentiation of melanoma | 1 |
| C.2.2.7 | Atypical variants of melanoma | 1 |
| C.2.2.8 | Special forms of skin cancer/tumors | 1 |
| C.2.3 | Feedback from dermatologist after referral | 5 |
| C.2.4 | Therapy | 3 |
| C.2.5 | Visual diagnostics | 2 |
| C.2.6 | Indication for excision | 2 |
| C.2.7 | Dermatological diseases in general / disease patterns | 1 |
| C.2.8 | Dermato-oncology in general | 1 |
| C.2.9 | Image teaching material | 1 |
| C.2.10 | Current information | 1 |
| C.2.11 | Aspects related to Covid-19 | 1 |
| C.2.12 | More knowledge in general | 1 |
| C.2.13 | Prevention | 1 |
| **Category 3: Implementation of SCS** | | |
| **Code** | **Subcategory** | **Frequency** |
| C.3.1 | Support by AI / digital tools | 4 |
| C.3.2 | Encourage self-examination of the skin | 1 |
| **Category 4: Training on SCS** | | |
| **Code** | **Subcategory** | **Frequency** |
| C4.1 | Expanding the training offer | 10 |
| C4.2 | Dermatoscopy course useful | 5 |
| C4.3 | Learning through job shadowing/practical exchange | 3 |
| C4.4 | Digital training useful | 1 |
| C4.5 | Training quality insufficient | 1 |
| C4.6 | Regular participation in training | 1 |
| C4.7 | Too much time required | 1 |
| C4.8 | No focus on statistics/indicators desired | 1 |
| **Category 5: Structural and organizational requirements of SCS** | | |
| **Code** | **Category** | **Frequency** |
| C5.1 | Combine screening with other examinations | 8 |
| C5.2 | Remuneration | 7 |
| C5.2.1 | Inadequate remuneration | 6 |
| C5.2.2 | Adequate remuneration | 1 |
| C5.3 | No timely appointments with dermatologist | 4 |
| C5.4 | Examination of genital area by gynecologist/urologist | 3 |
| C5.5 | Lack of cooperation of dermatologists | 3 |
| C5.6 | Quality of HKS at dermatologist insufficient | 2 |
| C5.7 | Utilization of skin cancer screening low | 2 |
| C5.8 | Dissemination of relevant information by dermatologist useful | 1 |
| C5.9 | SCS as an age-independent service makes sense | 1 |
| C5.10 | Lack of risk screening by dermatologist | 1 |
| C5.11 | Annual skin cancer screening desirable | 1 |
| C5.12 | Uncertainty in patients increased by dermatologists | 1 |
| C5.13 | Responsibility | 2 |
| **Category 6: Evaluation of SCS** | | |
| **Code** | **Subcategory** | **Frequency** |
| C6.1 | Low benefit rating | 1 |
| C6.2 | High benefit rating | 1 |
| C6.3 | Effectiveness questionable due to lack of risk screening | 1 |
| C6.4 | Implement assessment | 1 |
| C6.5 | False-positive findings due to nationwide screening < 25 years of age | 1 |
