# Supplementary material for: General practitioners’ perspectives on statutory skin cancer screening–A questionnaire-based cross-sectional survey in Germany
Source: PLoS One. 2024 Aug 8;19(8):e0308508. doi: 10.1371/journal.pone.0308508 (PMC11309404; doi:10.1371/journal.pone.0308508)
Supplement: Attachment — Submitted filename: PONE-D-23-14507.docx [file pone.0308508.s005.docx]

**Review Manuscript No. PONE-D-23-14507 entitled “General practitioners' perspectives on statutory skin cancer screening – a questionnaire-based cross-sectional survey in Germany”**

This is a well-written manuscript on the perspective of skin cancer prevention programs, which are essentially carried and implemented by primary care physicians. It can be assumed that due to the high quantitative demand the care of the people would not be guaranteed by the dermatologists alone. Already in 2014, a Schäfer et al. showed that tumor thickness is significantly higher in rural areas, in areas where specialist care is critical and will be increasingly so (1). Against this background, the present work is of political interest even if the geographical area as well as the participation is limited, but should emphasize this importance even more.

**Introduction**

- Are there any data about the number of performed screenings in Saxony and Bavaria by either general practitioners or dermatologists? This would be an interesting information.

**Methods**

Methods and statistics are described properly.

**Discussion and conclusion**

- The low participation of physicians has already been mentioned, and presumably physicians critical of screening in particular did not participate. This should be taken into account.
- Where possible, data on the frequency of screening in the two states should be related to the physicians surveyed and their proportion of screenings**.**

In summary, the paper addresses the perspective of general practitioners towards Skin Cancer Screening. Due to the geographic selection of two metropolitan regions, this is not a fully representative sample, but it nevertheless provides interesting information about the perspective of general practitioners.

(1) Schäfer I, Reusch M, Siebert S, Spehr C, Augustin M: Versorgungsmerkmale des Basalzellkarzinoms in Deutschland: Die Rolle von Versichertenstatus und sozio-demographischen Faktoren. J Dtsch Dermatol Ges. 2014
