# Supplementary material for: General practitioners’ perspectives on statutory skin cancer screening–A questionnaire-based cross-sectional survey in Germany
Source: PLoS One. 2024 Aug 8;19(8):e0308508. doi: 10.1371/journal.pone.0308508 (PMC11309404; doi:10.1371/journal.pone.0308508)
Supplement: Attachment — Submitted filename: Response_to_Reviewers.docx [file pone.0308508.s006.docx]

Felix G. Rebitschek

Academic Editor

PLOS ONE

**Ref.: PONE-D-23-14507**

**General practitioners' perspectives on statutory skin cancer screening – a questionnaire-based cross-sectional survey in Germany**

Universitätsklinikum Carl Gustav Carus · 01304 Dresden

Lydia Reinhardt

Telefon: 0351 458 19306

Lydia.reinhardt@ukdd.de

Dresden, den 25. Oktober 2023

Dear Mr. Rebitschek, dear reviewer,

Thank you very much for the valuable comments and the opportunity to improve and revise our manuscript. Below you will find our answers.

We have tried to address all the reviewers’ concerns in a proper way and believe that our paper has improved considerably. We would be happy to make further corrections if necessary and look forward to hearing from you soon.

On behalf of all co-authors sincerely yours,

Lydia Reinhardt

**Reviewer #1:**

1.1 Thank you very much for this interesting work on skin cancer screening. Considering the GPs perspective on the German statutory skin cancer screening is an important topic in order to also be able to assess the quality of the screening and derive future recommendations. Therefore, I rate the presented study positively, but think that it needs some major revision before publication. Overall the paper remains facile, specially in discussion and conclusion and the following comments should be considered
- Answer 1.1: Thank you very much for your valuable feedback and detailed comments, which we address below.
  1.2 Information on the validity of the study should be provided. Even though representativeness is not sought, comments on needed power and sample size would be good.
- Answer 1.2: Thank you for noting this. We have added comments in the Strengths and Limitations section, noting content validity, construct validity, and criterion validity, as well as the limited representativeness of the study (p. 16f.). Needed power and sample size were described in the Methods section, Quantitative analysis (p. 5f.).
  1.3 For assessing the informative value of the study, providing numbers of the total population in terms of age, gender and, if possible, years of experience for both regions to relate them to the study participants would be necessary. In addition, it would be important to know whether the characteristics mentioned differ between the two study regions in Bavaria and Saxony.
- Answer 1.3: We agree that this would be important to know. Unfortunately, we do not have additional information on age and professional experience of the general practitioners. From the Associations of Statutory Health Insurance Physicians (Kassenärztliche Vereinigung) in Bavaria and Saxony, we have only received the addresses of the general practitioners authorized to practice SCS. For data protection reasons, they did not give us further information on the physicians.

We have added the proportion by gender in the text (Methods, Participants section, p. 5).

1.4 The limited statistical representativeness should also be discussed.
- Answer 1.4: We agree and added a comment in the Strengths and Limitations section (please see also answer 1.2; p. 16).
  1.5 In the subgroup comparisons, the mean ranks should be given.
- Answer 1.5: We embedded the way the mean rank was calculated in the methodology section (p. 6) and then added the mean ranks in the subgroup analyses section next to the p-value (p. 10).
  1.6 The qualitative results presented appear to be more of a summary of the comments mentioned and not really a dedicated content analysis. A revision should take place here.
- Answer 1.6: We acknowledge that the purpose of the qualitative part may not have been clearly described. Therefore, we have specified our goal of qualitative analysis in the text, section “Results, Qualitative Analysis” (p. 10). The aim of our qualitative analysis was to categorize all the comments in the open questions (see Appendix S2, Questionnaire), to assign them to the topics of the survey and to identify additional and relevant aspects. Since the material is very extensive, we have only presented the most important main categories with example citations in the text. The full list of categories can be found in the appendix (S3).
  1.7 The discussion should be revised and be more comprehensively. It would be very useful to compare the results of skin cancer screening from the patient's point of view with the subjective perception of the GPs considering current studies on this (see e.g. Görig et al 2021).
- Answer 1.7: We appreciate your suggestion and hope that our additions have made the discussion more detailed. We refer to results on patient-reported quality of SCS (Görig et al., source 18). Where we have compared our results with the patients' view in the discussion, we have explicitly emphasized this (p.14).
  1.8 Overall, the significant results of the subgroup differences remain unconsidered.
- Answer 1.8: We acknowledge that our interpretation of the subgroup analysis remains vague, but we think the statistically significant results of our subgroup analysis should be interpreted with caution due to the small sample size. Further research with a larger sample would be desirable here. We have added this comment in the discussion (p.13f.).
  1.9 Since the 8-hour training is mentioned several times, some background information should be provided. For example, that the GPs and dermatologists complete the same training, that the content of the training is determined by the KFR and what content is taught there.
- Answer 1.9: Thank you for this suggestion. We added basic background information on the training and its content in the introduction section (p. 3).
  1.10 In addition, the conclusion demands that uncertainties and the use of a dermatoscope should be addressed in the training, which is both already part of the training, as far as I know. In addition, a refresher course and an evaluation are recommended. Such an evaluation has always existed and there was also a refresher, but it was discontinued due to low demand. Therefore, a mandatory refresher specified by the KFE may be more useful to be discussed.

Answer 1:10: Thank you for this valuable suggestion. We agree that both topics are already part of the training. However, in our survey, general practitioners made multiple comments on their uncertainty in the use of the dermatoscope, suggesting that these uncertainties were not adequately addressed or that the one-time training was not sufficient (we refer here exclusively to the comments in the open-ended questions). This is an important finding of our study.

We suggest a discussion of special courses for general practitioners or for subgroups like young professionals. However, since this was not the main question we investigated here, we refer to our publication on the the need for regular training in skin cancer screening (p. 16).

*Steeb T, Reinhardt L, Strasser C, Wessely A, Schelling J, Petzold A, et al. The need for regular training in skin cancer screening: a cross-sectional study among general practitioners in Germany. J Eur Acad Dermatol Venereol. 2022; 36:e913-e915. Epub 2022/07/06. doi: 10.1111/jdv.18366 PMID: 35757900.*

**Reviewer #2:**

This is a well-written manuscript on the perspective of skin cancer prevention programs, which are essentially carried and implemented by primary care physicians. It can be assumed that due to the high quantitative demand the care of the people would not be guaranteed by the dermatologists alone. Already in 2014, a Schäfer et al. showed that tumor thickness is significantly higher in rural areas, in areas where specialist care is critical and will be increasingly so (1). Against this background, the present work is of political interest even if the geographical area as well as the participation is limited, but should emphasize this importance even more.

- Thank you very much for your positive feedback.

Introduction

2.1 Are there any data about the number of performed screenings in Saxony and Bavaria by either general practitioners or dermatologists? This would be an interesting information.
- Answer 2.1: We agree that this would be an interesting information. We have added data in the Introduction on billed SCS from dermatologists and GPs in Saxony and Bavaria (2018), but point out that these data do not represent the actual number of screenings, as other examinations are also billed under SCS (p. 3f.).

Discussion and conclusion

2.2 The low participation of physicians has already been mentioned, and presumably physicians critical of screening in particular did not participate. This should be taken into account.
- Answer 2.2: Thank you for this important annotation. We added this in the Strength and Limitations section (p.16).
  2.3 Where possible, data on the frequency of screening in the two states should be related to the physicians surveyed and their proportion of screenings.
- Answer 2.3: Unfortunately, we only asked for the estimated number of screenings per month in our survey (“How many skin cancer screenings do you personally estimate you carry out each month? <5 / 5-10 / >10”). Therefore, this information cannot be compared with the absolute numbers.

In general, it is difficult to determine the actual number of screenings performed, as other examinations are also coded under this billing number – for example, when skin cancer is already suspected (please see Stang et al.).

*Stang A et al. The impossibility of mortality evaluation of skin cancer screening in Germany based on health insurance data: a case-control study. Eur J Cancer. 2022 Sep;173:52-58. doi: 10.1016/j.ejca.2022.06.009. Epub 2022 Jul 18. PMID: 35863106.*

2.4 In summary, the paper addresses the perspective of general practitioners towards Skin Cancer Screening. Due to the geographic selection of two metropolitan regions, this is not a fully representative sample, but it nevertheless provides interesting information about the perspective of general practitioners.
- Answer 2.4: Thank you for this appreciation!

**Additional Comment**

a. We added 4 new references (5, 7, 15, 16), which are highlighted in yellow in the reference list (unfortunately, our reference management software did not work with the track-change mode).
b. Please note that we also corrected two false p-values (typo) in the subgroup analysis section (p. 5) and highlighted them in the track-change mode.
c. We also added a new e-Mail address for the corresponding author on the title page.
