# Supplementary material for: General practitioners’ perspectives on statutory skin cancer screening–A questionnaire-based cross-sectional survey in Germany
Source: PLoS One. 2024 Aug 8;19(8):e0308508. doi: 10.1371/journal.pone.0308508 (PMC11309404; doi:10.1371/journal.pone.0308508)
Supplement: Attachment — Submitted filename: Response_to_Reviewers.docx [file pone.0308508.s007.docx]

Felix G. Rebitschek

Academic Editor

PLOS ONE

**Ref.: PONE-D-23-14507R1**

**General practitioners' perspectives on statutory skin cancer screening – a questionnaire-based cross-sectional survey in Germany**

Universitätsklinikum Carl Gustav Carus · 01304 Dresden

Cristin Strasser

cristin.strasser@ukdd.de

Dresden, den 25. März 2024

Dear Mr. Rebitschek, dear reviewer,

We would like to express our gratitude to you for providing us with valuable feedback. We have made every attempt to fully address your comments.

We hope that these revisions meet the requirements for publication in the PLOS ONE Journal. Please let us know if any further changes are necessary.

On behalf of all co-authors sincerely yours,

Cristin Strasser

**Reviewer #1:**

Thank you very much for your response and the consideration of my comments. They have all been addressed adequatly.

- Answer: We would like to thank you for your insightful comments. They were very helpful in improving the quality of our manuscript.

**Reviewer #3:**

I am coming to this manuscript as a new reviewer following revision by the authors. The authors appear to have addressed the comments from the previous reviews in a thorough and satisfactory manner.

- Answer: Thank you for this appreciation!

I have one minor comment, which is that it would be helpful to understand whether the training explicitly addresses which areas of the body should be screened for skin cancer. The authors highlight their findings about which areas of the body are less frequently screened by survey respondents, but they also mention that skin cancer is less frequent in those areas. It would be useful to understand the degree to which this is part of the training.

- Answer: Thank you for pointing this out. We have added information to clarify which areas of the body are explicitly addressed during training. Although we agree that information on frequency would be important, it cannot be provided in this manuscript because there is no detailed information available. However, we have mentioned that frequency of skin cancer is part of the training content.

The revised text reads as follows on page 3, lines 64-66:

„This training includes information on benefits and harms of early detection measures, clinical pictures and etiology, frequency and risk factors of skin cancer. The training is designed to ensure that physicians perform standardized visual whole-body skin examination, including scalp and intertriginous areas.“

My other comment is on data availability. The authors mention have made the survey instrument and categorization scheme available, but this is insufficient to reproduce the results. If it is possible to make an anonymized file of survey responses available, the authors should do so, or explain the restrictions that don't allow them to make this data available.

- Answer: We agree that this would be a potential limitation. Therefore, we have included the dataset in the Supporting Information.

**Additional Editor Comments:**

Thank you very much for your submission with important results about how GPs inform patients and how they conduct dermatology standard examinations in the real world, and for addressing the reviews!

A couple of issues yet need more elaboration. The following refers to the revision with track changes:

- Answer: Thank you very much for your detailed and useful comments. We have addressed each of them as follows.

1. Major
   1.1. It is not clear why you study what you study. Please show how you derive

- why you study, how the GPs perform/conduct the screening sessions, and, similarly,
- why you study their patient information strategies (what is here the desirable norm for comparison, are there deviations from informed screening decision making)?
- why you study their attitudes (evaluation)?
- Answer: Many thanks for this highly relevant comment. We have revised the introduction according to your recommendation (page 4, lines 86-94). In the Introduction, we present the rationale and motivation for our study in more detail, taking into account additional references. GPs are the most important supporters of skin cancer screening to detect skin cancer early. In particular, the studies by Tatiana Görig et al (DOI: [10.1111/phpp.12681](https://doi.org/10.1111/phpp.12681)) and Maleka Najmi et al (DOI: [10.1007/s00403-021-02224-z](https://doi.org/10.1007/s00403-021-02224-z)) explain the need to study the implementation of skin cancer screening in real life and understand the barriers and needs for acceptable and effective skin cancer screening practices from the GPs' perspective.
  1.2. The method leaves lacks that need to be filled for some reproducibility.
- It is very good that the questionnaire is attached. The section “survey”, typically “Measures”, should nevertheless explain better what they different parts are about. This should be comprehensible without the appendix. Was this instrument validated, or used before? Was it pre-tested? If not, how it was developed? Also, how were qualitative data measured?
- Answer: We have revised the text to address your concerns and hope that it is now clearer. Please see page 6 of the revised manuscript, lines 128-146 and page 7, line 147-148.
- How did you recruit with the help of the contact list (mailings)? How it was conducted (paper-and-pencil)? Was the procedure fixed, or could they scroll over the full survey back and forth?
- Answer: Please see page 5 of the revised manuscript, lines 116-118.
- Qualitative analysis plan is underspecified; for instance I cannot infer the measures for assuring quality; please check PLOSONE here: Qualitative research studies should be reported in accordance to the Consolidated criteria for reporting qualitative research (COREQ) checklist or Standards for reporting qualitative research (SRQR) checklist.
- Answer: We would like to point out that this is not an independent qualitative study, but rather a qualitative content analysis of the open questions in the questionnaire. The open questions were intended to obtain additional views and opinions from participants that may not have been captured in the questionnaire. Therefore, the suggested reporting guidelines are not applicable. Where possible, we have added information on the research process. To avoid further misunderstandings, we have removed misleading wording (“mixed-method”) and renamed "qualitative analysis" to "open question analysis".
- Because it is a survey study that aims to represent experts in Germany, we should get an indication of
  - How successful was recruitment, given population; recruitment and eligibility flowchart would be very helpful.
  - How representative was your sample (table 1), compared with the population of GPs in those states, or at least, in Germany, e.g. in terms of gender (an additional column with those data would be useful)?
- Answer: Thank you for this suggestion. We understand the rationale of providing information on recruitment and representativeness of our sample. Therefore, we have added a recruitment flowchart (see page 6, line 124). As we stated in our manuscript, all GPs with permission to perform SCS in the two regions were obtained from the Associations of Statutory Health Insurance Physicians in Saxony and Bavaria (December 2020). All physicians practicing general medicine at that time who were authorized to perform SCS and were active in the study region were included. The response rate was low, with 17.7%; please see page 5, line 120-123, and page 8, line 181. An analysis of the demography of physicians or the population in general was not part of our analysis and therefore we cannot make a validated statement on this topic.
  1.3. One can stress that the key results lie in the compliance with how to inform patients prior to examination (e.g., guidelines on evidence-based health communication) and how to conduct skin cancer examinations for purpose of screening (e.g., dermatology guidelines). Accordingly,
- You could also change Figure 1 to the analysis of proportions (I always…) This would be consistent to Figure 2 and supportive to the function of a visual (“patterns”) – complex ranking data as here could go with a table.
- Answer: We have modified the figure as suggested.
- Calculate (particularly with regard to further subgroup analysis) the proportions of those, who do not fully inform, and those who do not fully conduct. This stresses the need for intervention as you discuss it already.
- Answer: We thank the reviewer for the valuable suggestion, and we have incorporated the proposed proportions into the article (please see page 10, lines 199-200 and lines 221-222). The proportion of those, who do not fully inform is 95.1% (194 out of 204). Only ten GPs always provide comprehensive information in the context of SCS. The proportion of those, who do not fully conduct the SCS is 68.1% (139 out of 204). Only 31.9% (65 out of 204 GPs) always performed the skin cancer screening in its entirety.
  1.4. Subgroups: Because there was no preregistration and particularly no statistical hypotheses, please adjust alpha for multiple testing (depending on the number of your comparisons there). Furthermore, I would suggest to conduct those subgroup analyses preferably with the proportion of those who “always” did something (more robust!)
- Answer: We extend our gratitude to the reviewer for highlighting the importance of adjusting alpha due to multiple testing. In response, we have performed the Bonferroni correction, resulting in a significance level of alpha=0.05/6=0.0083 in the subgroup analysis (see page 11, lines 226-227). Additionally, we appreciate your suggestion to focus on individuals who 'always' did something. Subsequently, we conducted Χ^2^ -tests for these specific groups. Altogether, we yield fewer significant findings, yet enhancing the robustness of our results.
  1.5. Check reporting of qualitative results against Consolidated criteria for reporting qualitative research (COREQ) checklist or Standards for reporting qualitative research (SRQR) checklist, e.g. how many said something “was described as not relevant”…?
- Answer: Please see our response to point 1.2 and 2.1. The full set of categories and their frequencies can be found in S4 Appendix.
  1.6. Discussion
- This should come back to the arguments that underlie your study motivation (see first point above).
- Answer: Many thanks for this suggestion for improvement. We start the discussion by presenting the rationale and motivation for our study on the basis of the study data available to date (page 14, lines 309-317 and page 15, lines 318-322). In the following discussion, our study data are discussed in detail in the context of the existing literature, including the studies that triggered the rationale for our study.

2. Minors
   2.1. Suggestion to generally keep the order of your results section across the manuscript, first writing about “Informing patients” and then “Performing screening” (as it would be at the GP’s office)

- Answer: Thank you for your suggestion. As many of the results of the open question analysis are not directly linked to the results of the quantitative analysis, we would like to continue to report them separately.
  2.2. Consistent capitalisation across tables and figures and their elements please
- Answer: Revised accordingly.
  2.3. Consistent use of SCS across Figure captions and manuscript
- Answer: Revised accordingly.
  2.4. There are very important statements which do not have a reference, but which are result of the study, nor general knowledge
- L.53,54
- L.61/62
- L. 263/264
- L. 269 – could you check real-world materials for this aspect?
- Answer: Thank you. We have added the missing references.

Regarding your question on line 269 (now line 329): The training content is standardized according to the guideline for early cancer detection and includes information on harms of SCS. Nevertheless, the training content could be taught differently depending on the training provider. Unfortunately, these training materials are not available publicly.

2.5. Abstract
- available = available free of charge?
- Answer: We have added “free of charge”.
- Objective: How = how comprehensive
- Answer: We have added “comprehensively”.
- What other issues… This needs to be more concrete. What did you want to find out? How they inform patients?
- Answer: We have changed the sentence.
  2.6. Introduction:
- Do ref 2,3 control for screening programme introductions?

Answer: We are not sure whether we have understood the question correctly. Both references refer to the rising skin cancer incidence and risk factors, and to screening as a part of secondary prevention of skin cancer.

- Please add a sentence (based on the best available evidence) about benefits and harms of skin cancer screening for the general population from 35 years
- Answer: Please see page 3, lines 72-73 and page 4, line 74.
- L.70-73, please provide the baseline numbers (of GPs and dermatologists)
- Answer: We have added the suggested content to the manuscript. Please see page 4, lines 77-79.
  2.7. Methods
- Please check order of sections against PLOSONE guidelines; also study population could go to the Methods
- Answer: We have switched the order of sections where necessary.
- Funding statement comes separately
- Answer: The sentence has been deleted from the text.
- Delta is between absolute means, if so, expected effect size? To which outcome was this expectation referred?
- Answer: We conducted the sample size calculation with the hypothesis H_0_: "I feel adequately trained to perform skin cancer screening" (The mean of the survey is thus μ≤2) against H_1_: "I do not feel adequately trained to perform skin cancer screening" (μ>2). A delta of 0.5 was considered a plausible minimal effect size, which we aimed to detect with a power of 90%.
  Questions on the need for regular training in SCS have been published elsewhere (DOI: [10.1111/jdv.18366](https://doi.org/10.1111/jdv.18366)).
  2.8. Results
- L.160 , n=1?
- Answer: It should be “missing=1”. We have corrected this.
- For your quantitative sample-based results from which you would like to generalise in the discussion, you need to provide uncertainty information (e.g. confidence intervals)
- Answer: We appreciate the reviewer for bringing up this point. In response to your suggestion, we have modified the data analysis by conducting Χ^2^ -tests instead of the Mann-Whitney U test and the Kruskal-Wallis test. Consequently, these are now hypothesis tests that calculate the p-value but do not yield a confidence interval.
  2.9. Discussion
- L. 319 “in which the uncertainties…” could be skipped here, because this is only a sub-aspect
- Answer: We agree and have skipped this detail.
- The construct validity does not depend on the sample; explain why recall bias affect construct validity?
- Answer: Thank you for bringing this to our attention. The revised text reads as follows on page 19, lines 417-419: “The survey might be biased by the selection of the sample and the memories of the participants (recall bias).”

**Additional Comment**

a. Please note that the corresponding author has changed. We have added a new e-mail address for the corresponding author on the title page.
