# Supplementary material for: General practitioners’ perspectives on statutory skin cancer screening–A questionnaire-based cross-sectional survey in Germany
Source: PLoS One. 2024 Aug 8;19(8):e0308508. doi: 10.1371/journal.pone.0308508 (PMC11309404; doi:10.1371/journal.pone.0308508)
Supplement: Attachment — Submitted filename: Response_to_Reviewers.docx [file pone.0308508.s008.docx]

Felix G. Rebitschek

Academic Editor

PLOS ONE

**Ref.: PONE-D-23-14507R2**

**General practitioners' perspectives on statutory skin cancer screening – a questionnaire-based cross-sectional survey in Germany**

Universitätsklinikum Carl Gustav Carus · 01304 Dresden

Cristin Strasser

cristin.strasser@ukdd.de

Dresden, den 26. Mai 2024

Dear Mr. Rebitschek, dear reviewer,

We would like to thank you for your constructive and detailed comments on our manuscript. The recommendations and advice have helped us to improve the quality of the manuscript significantly.

As suggested, we have revised Figures 2 and 3. We hope that our resubmission is now suitable for inclusion in PLOS ONE and we look forward to hearing from you.

On behalf of all co-authors sincerely yours,

Cristin Strasser

**Additional Editor Comments:**
Thank you for your substantial and detailed revision!

- Answer: Thank you for your appreciation!

One point remains concerning the Figures 2 and 3. As I pointed out before about text. If the authors want to report conclusions about the population of GPs practising SCS, sample-based data should not be reported without uncertainty estimates in the figures. Without those clarifications figures could be easily misinterpreted.
If this is addressed, the manuscript can be published.

- Answer: We thank you for this valuable suggestion and agree that it is important to include uncertainty estimates in the figures. We have calculated the 95 % confidence intervals. Figures 2 and 3 now include error bars.
